# Implementation of an Australian helpline for low back pain: protocol of a type 2 hybrid effectiveness-implementation trial

**DOI:** 10.1136/bmjopen-2025-106605

**Published:** 2025-12-02

**Authors:** James Zouch, Katharine Roberts, Adrian Bauman, Helen Jentz, Emma K Ho, Paul Hodges, Chris Maher, Melissa T Baysari, Julia Thompson, Rosemary Calder, Georgina Luscombe, Dragana Ceprnja, Katherine Maka, Ye Tian, Yanyu Chen, Michelle Chen, Paul Jarle Mork, Qiang Li, Sarah Wise, Melissa Gilbert, Michelle Hall, Manuela L Ferreira, Paulo Ferreira

**Affiliations:** 1 Sydney Musculoskeletal Health, The University of Sydney Faculty of Medicine and Health, Sydney, New South Wales, Australia; 2 Charles Perkins Centre, School of Public Health, University of Sydney, Sydney, New South Wales, Australia; 3 Musculoskeletal Health Australia, Melbourne, Victoria, Australia; 4 Centre for Innovation in Pain and Health Research, The University of Queensland Faculty of Health and Behavioural Sciences, Brisbane, Queensland, Australia; 5 Sydney Musculoskeletal Health, The University of Sydney, Sydney, New South Wales, Australia; 6 Institute for Musculoskeletal Health, Sydney Local Health District, Camperdown, New South Wales, Australia; 7 The University of Sydney Faculty of Medicine and Health, Sydney, New South Wales, Australia; 8 New South Wales Agency for Clinical Innovation, St Leonards, New South Wales, Australia; 9 Victoria University Institute for Health and Sport, Melbourne, Victoria, Australia; 10 School of Rural Health, Sydney Musculoskeletal Health, The University of Sydney Faculty of Medicine and Health, Sydney, New South Wales, Australia; 11 Department of Physiotherapy, Westmead Hospital, Westmead, New South Wales, Australia; 12 Virtual Care and Hospital in the Home Services, Northern Sydney Local Health District, St Leonards, New South Wales, Australia; 13 Department of Public Health and Nursing, Norges teknisk-naturvitenskapelige universitet, Trondheim, Norway; 14 The George Institute for Global Health, Sydney, New South Wales, Australia; 15 School of Public Health, University of Technology Sydney, Sydney, New South Wales, Australia; 16 Olga Tennison Autism Research Centre, School of Psychology and Public Health, La Trobe University, Bundoora, Victoria, Australia; 17 Musculoskeletal Pain Hub, Charles Perkins Centre, Kolling Institute, The University of Sydney Faculty of Medicine and Health, Sydney, New South Wales, Australia; 18 Musculoskeletal Pain Hub, Charles Perkins Centre, Sydney Musculoskeletal Health, The University of Sydney Faculty of Medicine and Health, Sydney, New South Wales, Australia

**Keywords:** Primary Health Care, Self-Management, Musculoskeletal disorders, Back pain, Health Services, Implementation Science

## Abstract

**Introduction:**

Low back pain (LBP) is the leading contributor to disability globally. It has a substantial impact on the lives of those who experience it, and places considerable economic burden on healthcare systems. Despite these impacts, and the consistency of guideline recommendations, many individuals do not receive recommended LBP management. Structural barriers to accessing timely, evidence-based care, as well as public uncertainty about where to seek appropriate management, can influence the care individuals receive. Telephone and digitally based helplines assist to overcome many traditional barriers to accessing care and offer a scalable platform to improve the delivery of guideline recommended management for LBP. However, uptake of such services can be limited without targeted promotion and patient-centred design. This project aims to codesign, implement and evaluate an upgraded component of an existing Australian helpline service, tailored for people with back pain and supported by a media awareness campaign. This protocol outlines the codesign process, implementation and planned evaluation of the helpline.

**Methods and analyses:**

This protocol uses three complementary frameworks—an iterative codesign process, the Practical Robust Implementation Sustainability Model, and the Reach, Effectiveness, Adoption, Implementation and Maintenance framework—to guide the codesign and development, implementation and evaluation of an upgraded helpline for people with LBP. The codesign process involves key stakeholders, including consumers and clinicians, to inform the development and implementation of both the upgraded helpline service and the media campaign to raise awareness and uptake of the helpline. Data sources will include a pre–post cohort of helpline service users, routinely collected service data (eg, monthly call rate) and health system data to evaluate the broader population level impact (eg, rates of emergency department presentations for LBP in the Australian region targeted by the media campaign). Implementation evaluation will include Reach, Effectiveness, Adoption, Implementation and Maintenance as well as internal and external environmental factors that influence the success of these outcome measures.

**Ethics and dissemination:**

The project was approved by the University of Sydney’s Human Research Ethics Committee (HE001081). This project involves collaboration with consumers, clinicians and other stakeholders to interpret, translate and disseminate research findings to relevant audiences.

**STRENGTHS AND LIMITATIONS OF THIS STUDY**

- This study uses a multiframework approach (iterative codesign, Practical Robust Implementation Sustainability Model and Reach, Effectiveness, Adoption, Implementation and Maintenance) to provide a transparent and theory-informed structure for the design, implementation and evaluation of the upgraded helpline service.
- Extensive patient and public involvement is embedded throughout the study via the codesign, implementation and evaluation processes with consumers, clinicians and other stakeholders.
- Multimethods evaluation using quantitative service and cohort data with qualitative insights from stakeholders will provide an improved understanding of potential barriers and facilitators to successful implementation.
- The use of a pre–post cohort design allows comparison of service effectiveness before and after the upgrade; however, in the absence of a randomised control group, causal inferences are limited.
- Cohort participants are anticipated to be primarily from the geographical region targeted by the media campaign, which may limit generalisability to other regions with different health service and demographic profiles.

## Introduction

 Despite efforts to change policy^1 2^ and calls to improve practice,^3 4^ low back pain (LBP) remains the leading cause of disability, both in Australia and globally.^5^ LBP has an estimated mean point prevalence of 18%^6^ with a high recurrence rate^7^ and an unfavourable clinical course for those who develop chronic symptoms.^8^ Additionally, LBP is associated with financial hardship and poverty and is the leading chronic condition associated with early workforce retirement.^9 10^ Thus, the economic burden associated with back pain (of which LBP is the largest contributor) in Australia is substantial. Direct health system costs are estimated at $A3.4 billion annually^11^ and indirect costs, associated with loss of productivity and increased welfare payments, exceed $A6 billion.^11^

Guideline management recommendations for LBP have remained relatively consistent for 20 years.^12^ They advocate for a non-pharmacological approach that includes advice and education, self-care and physical activity. Imaging is recommended only for suspected serious pathology and medications should be used judiciously.^13^ However, evidence on contemporary LBP management practices indicates a gap between guideline-recommended care^13^ and the care that patients receive.^14^,^16^ For example, in primary (first-contact) care settings, approximately 25% of patients with LBP are referred for imaging,^16^ more than one-third are prescribed at least one opioid medication within a year of diagnosis,^17^ and only half of Australian patients surveyed recalled receiving education and advice.^18^ In emergency departments (EDs), nearly 70% of patients presenting with LBP receive an opioid medication and almost one-quarter are imaged.^19^ This discrepancy may reflect inequities in access to high-value care and resources, and inadequate support to self-care.

While considerable research has been directed toward improving clinician adherence to LBP guidelines,^20^ addressing structural barriers within the Australian healthcare system may be essential to bridging the gap between guideline-recommended LBP care, and the care that patients receive. Key barriers include inadequate access to timely and appropriate care,^21^ limited public funding to support allied health management^22^ and affordability of ‘out-of-pocket’ costs and private health insurance.^23^ Exacerbating these structural barriers are widespread public misconceptions about the causes of LBP and appropriate management choices. Combined with patients’ lack of awareness about the roles and scope of practice of different healthcare providers, this often results in unsupported self-care, uncertainty about where to seek appropriate care for management of their LBP,^21^ and unnecessary health service utilisation.

The evidence highlights an urgent need to explore alternative strategies to reduce the widespread impact of LBP on individuals and the healthcare system.^2^ Through reformed healthcare pathways and funding models, these strategies should focus on improving access to evidence-based information and care, supporting individuals to remain active at work and in daily life, and reducing unnecessary health service utilisation. One potential solution is the use of helplines—telephone-based services that typically provide free access to health information and advice, with the aim of reducing face-to-face care.^24^ They overcome traditional barriers to accessing care and provide a platform to disseminate evidence-based educational resources at scale and with little geographical limitation. Helplines have been used to empower individuals by providing tailored evidence-based information, emotional support and advice for those with mental health or substance use problems,^25 26^ chronic diseases such as cancer^27^ or rheumatological conditions.^28^ While several helplines exist in Australia to support individuals with musculoskeletal pain or related conditions, such as arthritis, currently no helpline offers a dedicated back pain service that has been evaluated for effectiveness. However, preliminary evidence suggests that LBP interventions delivered by telephone or digitally (eg, mobile apps, websites)^29 30^ can reduce pain intensity and disability when compared with usual care.^31^

Musculoskeletal Health Australia (MHA) is a national peak organisation that represents Australians living with musculoskeletal conditions and aims to support improved musculoskeletal health. MHA established a national helpline (https://muscha.org/helpline) in 2011 to provide free support to people living with musculoskeletal conditions. The helpline is delivered by allied health professionals with clinical experience in managing musculoskeletal pain. It operates from Monday to Friday from 9:00 to 17:00, excluding public holidays (typical business hours in Australia) and is accessible for callers requiring translation services or who have hearing or speaking difficulties. Currently, callers receive one-off, telephone-based support and information. Helpline operators predominantly assist callers with understanding musculoskeletal conditions, managing symptoms including pain, and navigating the complex healthcare system.

Building on this foundation and leveraging digital health technology—including telephone calls, video conferencing and digital health platforms—we will develop and evaluate an upgraded component of MHA’s existing helpline, specifically tailored to individuals experiencing LBP. The enhanced service will incorporate tailored education and advice, empower patients and support self-care, assist patients in navigating the healthcare system and provide follow-up support. The project also includes a media campaign to raise community awareness and encourage greater use of the upgraded helpline.

The aims of the project are:

1. Upgrade and tailor a component of the MHA helpline service specifically for people with LBP.
2. Implement the upgraded MHA helpline and promote it through a targeted and evaluated mass media campaign.
3. Evaluate outcomes of the service implementation: reach, adoption, effectiveness (including clinical and cost-effectiveness) and maintenance and identify factors contributing to the outcomes.
4. If proven effective, to develop, with our project partners, a plan for an Australia-wide deployment and sustainability of the upgraded MHA helpline.

## Methods

### Study design

This study will use an implementation-effectiveness hybrid design and is funded for the period 1 May 2024 to the 30 April 2029. The study protocol is guided by the Standards for Reporting Implementation Studies.^32^

To guide the design and implementation of the upgraded helpline, an iterative codesign approach, adapted from the Active Implementation Framework,^33^ will be employed.

This will involve iterative phases of engagement with a continuous feedback loop to key stakeholders, including clinicians and consumers and our research partners. This approach aims to ensure the upgraded service remains aligned with stakeholder objectives and the needs of end-users throughout the codesign and implementation process. The planned iterative phases will include:

1. Exploration—identifying existing issues with the current MHA helpline and establishing the needs of end-users.
2. Design and development—collaborative design of the upgraded helpline and resources needed to successfully implement the service.
3. Testing and refinement—piloting of the upgraded helpline and systems.
4. Implementation—full implementation of the upgraded service and marketing campaign.

**Figure 1:**
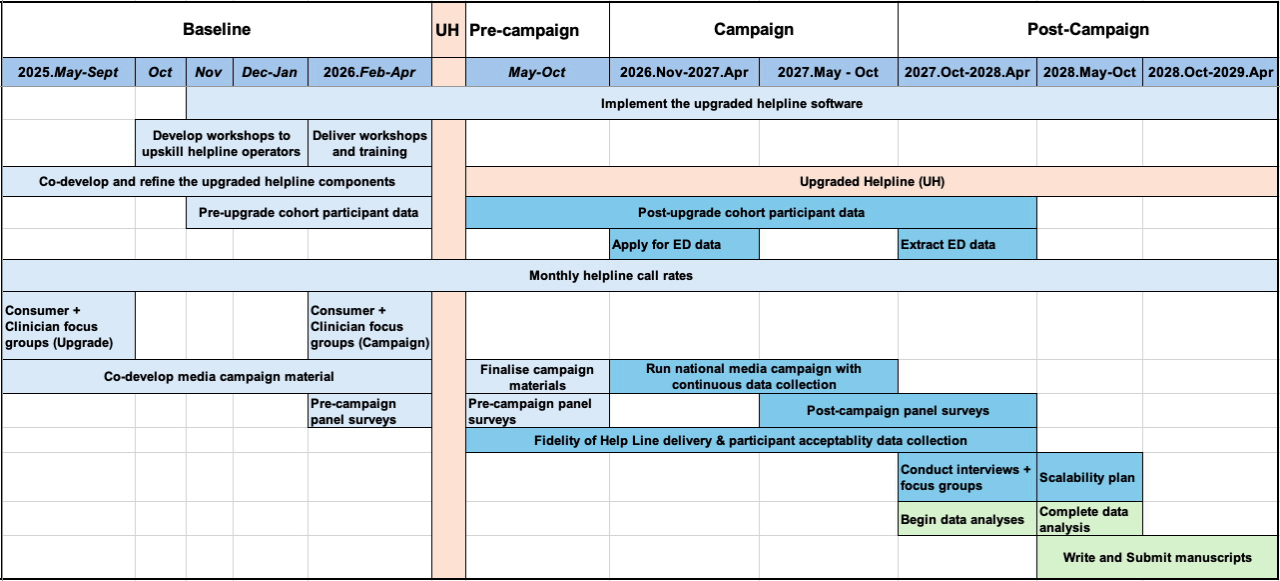
Project timeline illustrating key phases of data collection, helpline upgrade and media campaign activities, alongside related implementation processes. ED, emergency department; UH, upgraded Helpline.

The evaluation component will employ a mixed-methods approach to capture implementation process, service outcomes and user experience. Data collection will include: (1) codesign focus group with clinicians and consumers to inform service design and delivery, (2) targeted population surveys to determine helpline awareness before and after the media campaign, (3) pre–post cohort of helpline users to assess participant-reported outcomes, (4) qualitative interviews with a purposive sample of cohort participants and stakeholders to inform implementation and scalability and (5) aggregated data on LBP presentation rates to EDs within the campaign target area. See figure 1 for the project timeline.

### Setting and stakeholders

While the research team will assist with the codesign, implementation and evaluation of the upgraded LBP component of the service, the helpline will continue to be managed and operated by MHA. This free service is currently available to all Australians with telephone access. However, for the purposes of the evaluation, the proposed media campaign will be primarily delivered in the Mid North Coast region—a defined Local Health District, within the state of New South Wales (NSW). The Mid North Coast region has a population of 230 000 broadly reflective of national demographics.^34^ The region was chosen due to its concentrated local media which will enable cost-effective dissemination of media campaign messages and maximise the likelihood of helpline awareness and uptake of the service. Therefore, we anticipate most users of the helpline service and recruited cohort participants will be from that geographical area during the campaign period.

The research partners collaborating on this project include:

- MHA, primary research partner and helpline operator. MHA also has an associated consumer advisory committee that works closely with the MHA organisation.
- Agency for Clinical Innovation, a state-based agency working with consumers, clinicians and partnering with the state Ministry of Health and Local Health Districts to improve health systems within NSW, Australia.
- Australian Self-Care Alliance, a collaborative organisation of healthcare consumers, health promotion charities, policy experts and industry partners that aim to promote self-care among Australians.
- Northern Sydney Local Health District (NSLHD) Virtual Care and Hospital in the Home services NSLHD, which manages health facilities, including public hospitals, clinics and healthcare services, provided to the geographical region of Northern Sydney, NSW and also delivers a Virtual Care Service model. The NSLHD will provide expertise on remote care delivery.

### Patient and public involvement

Patients and the public have been actively engaged from the inception of this project and will continue to be engaged throughout the design, development, evaluation and dissemination, as outlined in this manuscript. Partnerships with consumer advocacy organisations (MHA and the Australia Self-Care Alliance) have helped identify the gaps in LBP service and will enable a collaborative approach to developing service solutions that address patient priorities. By using an iterative codesign process with ongoing feedback loops, this project aims to ensure the project development is aligned with the needs of all stakeholders, including patients, partner organisations, clinicians and the public. Partner organisations and participating consumers will contribute to the project evaluation, interpretation of results and dissemination of research findings.

### Participant recruitment and consent

#### Codesign consumer and clinician focus group participants

The codesign consumer group will be recruited through MHA’s consumer advisory committee and will include the research team’s consumer representative, as well as invited consumers from previous research projects. MHA’s consumer advisory committee and the research team will help identify and purposively recruit members to ensure representation across age groups and sex. The consumer group will include between 8 and 12 members.

The clinician group will include project members and invited clinicians recruited through professional networks of the research team. Recruitment will be purposive to ensure representation from both private and public sectors. The clinician group will include between 8 and 12 members. Eligible participants will be adult participants (≥18 years) who can communicate in English, have access to videoconferencing software (eg, zoom or Skype) and are available to attend an online meeting at a scheduled time and date. Invited participants will receive an electronic information sheet and provide consent electronically via an online REDCap (Research Electronic Data Capture) link, prior to participation. REDCap is a secure, web-based software platform designed to support data capture for research studies.^35^

#### Campaign awareness survey

Media campaign awareness will be assessed using a separate online panel survey administered by a commercial panel provider, with data that can be weighted to the regional population. The company will purposively recruit a sample of adults (≥18 years) residing in the Mid North Coast and residing in a comparison area, the Southern NSW Local Health District. A single campaign awareness question will be asked within existing panel surveys. This will allow us to determine campaign awareness in the exposed region and in a demographically similar region. Only aggregated, deidentified responses will be provided to the research team; therefore, individual informed consent is not required in accordance with institutional ethics approval.

#### Pre–post cohort study participants (from the helpline)

Two independent cohorts (prehelpline and posthelpline upgrade) will be recruited using consecutive sampling of helpline users presenting with a primary complaint of LBP. At the conclusion of their helpline call, helpline operators will verbally invite eligible users to participate in a research study evaluating the helpline service. Interested individuals will receive an electronic information sheet via an online (email or text) REDCap link. Eligible participants will be adults (≥18 years) who are able to provide informed consent for study participation and have sufficient understanding of written and spoken English to read and complete online forms, and have used the helpline service for advice/management of their LBP. Informed consent will be obtained electronically using REDCap’s e-signature feature, prior to research participation in accordance with the institutional ethics approval. Participants can only be enrolled once in the same research period (ie, in the preperiod or postperiod) but can participate in both. Consenting participants will receive an invitation to complete the baseline, 3-month and 6-month questionnaires via REDCap.

#### Cohort qualitative interviews

A purposive subsample of an estimated 15–20 (total) pre–post cohort participants will be invited to participate in individual qualitative interviews, to explore factors contributing to helpline uptake, user experience and contextual factors relevant to scalability. Sample size will be guided by data saturation and sampling approach designed to capture diverse perspectives across variables such as health confidence, helpline acceptability, age and LBP chronicity (completed as part of the participant-reported outcomes).

#### Stakeholder focus groups

Two stakeholder focus groups will be held as part of the process evaluation of the helpline: one with helpline operators and one with partnering health service providers (3–10 participants per group). Participants will be purposively selected to ensure a range of experiences are captured (eg, years of clinical experience, health profession). Eligible participants, adults (≥18 years), will be provided with the study information and consent forms electronically prior to attending an online meeting.

### Design

**Figure 2:**
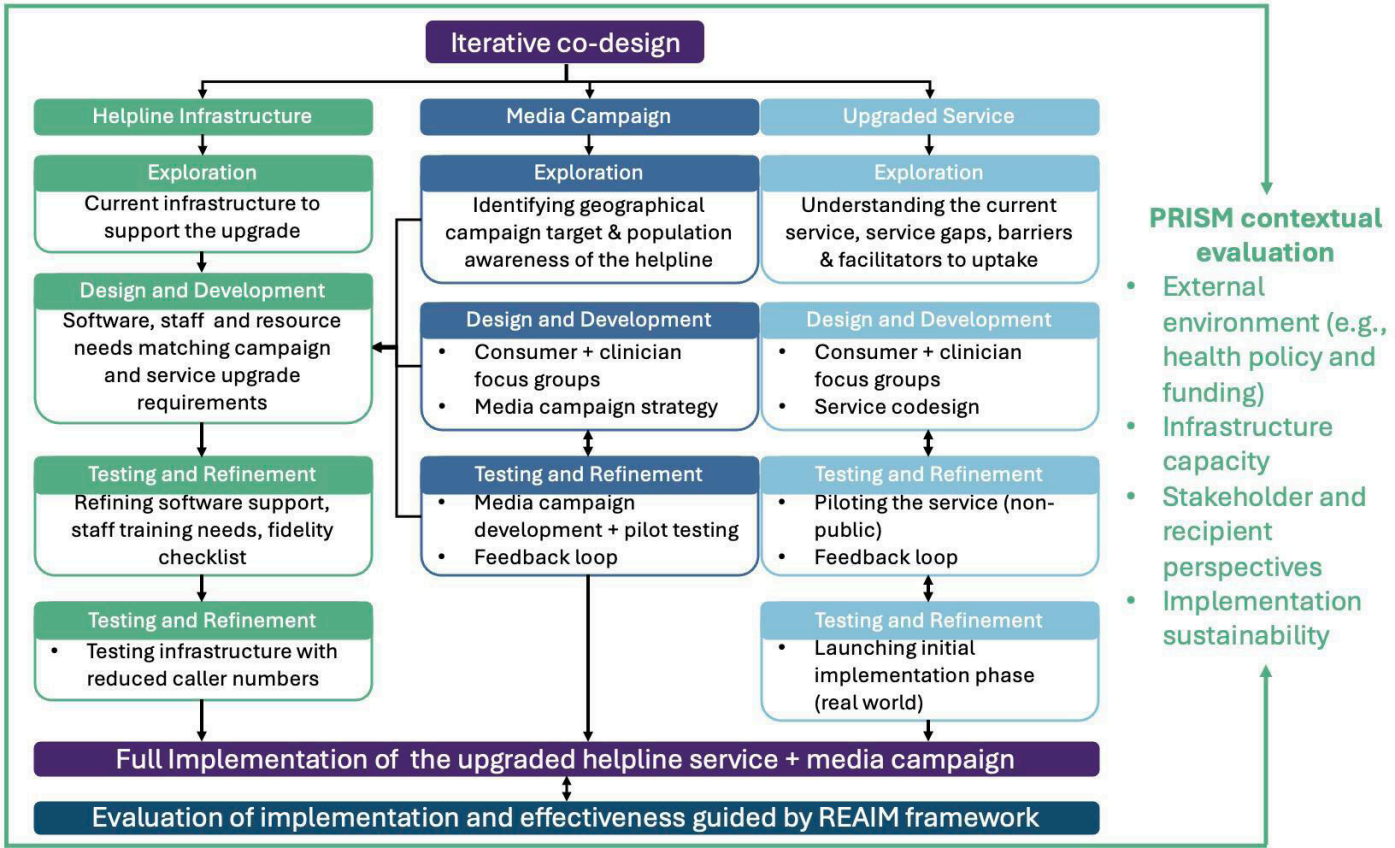
Project flow chart illustrating the iterative codesign and implementation process for the helpline infrastructure, media campaign and upgraded helpline service. Evaluation will be guided by the PRISM and RE-AIM frameworks. PRISM, Practical Robust Implementation Sustainability Model’ RE-AIM, Reach, Effectiveness, Adoption, Implementation and Maintenance.

This project uses three overarching frameworks: (1) an iterative codesign that describes the implementation process,^33^ (2) the Practical Robust Implementation Sustainability Model (PRISM)^36^ used to evaluate and understand the contextual factors that influence implementation and effectiveness outcomes and (3) the Reach, Effectiveness, Adoption, Implementation and Maintenance (RE-AIM) framework^37^ to evaluate the project implementation and effectiveness.^38^ Figure 2 depicts the project flow chart incorporating these frameworks.

#### Exploration

In this phase, project partner consultations with MHA’s organisational staff, helpline operators and their consumer advisory group will be conducted to understand current gaps in the helpline service and supporting systems, and potential barriers and facilitators to upgrading the service. Separate online group meetings will be conducted, one with available organisational staff (approximately 2–4 members) and one with the consumer advisory committee (approximately 5 members). These meetings will form part of existing partnership engagement agreements.

A review of MHA’s organisational needs, priorities and values will provide context for the design and suitability of the upgraded service in the design stages. This will be explored alongside a review of current helpline data, to provide a broader understanding of end-user needs and the resources and capacity of the helpline to meet those needs.

In addition, the research team and MHA, in consultation with a media agency, will identify suitable locations and strategies for the media campaign. This will consider the potential cost-effectiveness of advertising in each region, the available health services (eg, ED’s and urgent care clinics), and the representation of metropolitan, regional and rural locations.

#### Design and development

Codesign of the upgraded helpline service will be informed by focus groups conducted with two separate groups, one comprising consumers (potential helpline users) and the other comprising clinicians. The focus groups will be conducted separately to capture the distinct perspectives of each group. One online nominal group technique^39^ meeting (for each group) will be used to generate, discuss and prioritise the key components that should be included in the upgraded helpline design, addressing both consumer needs and clinician perspectives on what is appropriate and necessary to provide effective LBP management support. It is anticipated these meetings will take between 1.5 and 2 hours. The focus group findings will be incorporated into an initial design of the upgraded helpline, which will be presented to the research team, ensuring alignment with the project aims, MHA’s needs and a sustainable model of care as advocated by our partner organisations. This design will be refined through a feedback loop where focus group members will be invited to review the design via email and provide individual feedback, allowing further development.

The same focus groups and meeting structure will be used to codesign the targeted media campaign with the objective of raising awareness of the upgraded helpline service for LBP. Development of the media campaign will be guided by the key messages identified by consumers and clinicians to promote uptake of the upgraded service. Key campaign messages will be pilot tested with our research partners including MHA’s consumer advisory committee and refined before media content production. Key elements necessary for a successful media campaign (ie, consumer messaging, proportion of the target audience made aware of the campaign, target location and available resources, cost, long-term impact and sustainability) will be designed and developed in consultation with an externally contracted media agency.

The third component of the codesign phase will comprise the development of infrastructure needed to support the helpline. This will include the integration of software systems and resources to support the upgrade, and sustainable processes to monitor and evaluate the helpline. It will also involve outlining the roles and responsibilities of helpline operator staff to meet the increased demands of the upgraded helpline, employing new helpline staff and developing staff training to upskill operators in the delivery of the upgraded service and use of new systems. Fidelity checklists will be codesigned with helpline operators to evaluate delivery of the upgraded service and to identify changes needed to support ongoing delivery of the upgraded service.

### Testing and refinement

The upgraded helpline service and workflows will be tested through a two-step process: (1) A pilot phase with a small number of invited pilot users (non-public release). Pilot users will include members of our research partner organisations, project operations team and MHA’s consumer advisory group, ensuring representation from each partner. We anticipate a minimum of five users. Pilot users will complete the System Usability Scale^40^ to provide structured feedback on the usability of the helpline service. This process will be complemented by an online consultation with pilot users to explore the service functionality, ease of navigation and helpline operator communication. (2) Initial implementation trial and refinement 6 months prior to the media campaign. This will allow time to test the upgraded service in ‘real-world conditions’ prior to the media campaign and an anticipated increase in service users. It is estimated this will involve 50 participants based on MHA’s current monthly rate of LBP callers. These participants will be recruited consecutively following use of the helpline service (see the participant consent and recruitment section) and form the first 50 participants of the postupgrade cohort.

Cohort participants will rate their acceptability of the helpline service across several domains using a modified version of the Theoretical Framework of Acceptability Questionnaire (TFAQ).^41^ In addition, routine service information including the number of calls, call answer rates, peak call times, call duration, number of urgently referred cases, proportion of calls requiring follow-up and average follow-up calls will be collected in the initial implementation period (prior to the media campaign release).

This information will be combined to identify any operational, logistic or technical adaptations that need to be made prior to the full implementation stage.

### Implementation

Full implementation will encompass developments and adaptations of the upgraded service made during the testing-refinement phase. It will also involve the launch of the targeted media campaign. Importantly, this phase will incorporate findings from the project evaluation (outlined below) to further refine and develop the upgraded helpline service with respect to RE-AIM outcomes. The objective of this stage is to embed a process of ongoing evaluation that will support continued development and sustainability of the helpline service beyond the lifecycle of research funding.

### Media campaign

To increase reach, awareness, service use and potential impact of the upgraded helpline, a targeted mass media campaign will be conducted. Mass media campaigns use media sources such as radio, television and social media to disseminate information/messages to a large audience.^42^ Previous studies suggest that this method can effectively promote and increase calls to helpline services.^42^,^44^ The campaign messaging will be codesigned with consumers, the research team and a dedicated advertising agency to create a campaign strategy that aligns with the objectives of MHA’s helpline and the objectives of the partnership project. The media campaign content will be created in consultation with a professional content creation company and reviewed by the research team, partner organisations and consumers prior to launch.

### The tailored LBP component of the helpline service

The proposed model of care offered by the upgraded helpline will be centred around a ‘live telephone service’ providing an opportunity for individuals with LBP to share and discuss their LBP experience with a qualified healthcare professional. Importantly, the helpline will be a telephone and digitally based resource for people with LBP to help make sense of their pain, to provide information and advice to understand their management options and assist navigating the healthcare system and to support self-care. These core concepts align with patients’ perceived needs from a healthcare service for managing musculoskeletal pain,^45^,^48^ and the Australian Low Back Pain Clinical Care Standards.^13^ It is anticipated that the service will also include a screening and triage component to identify callers with suspected serious pathology. During triage, helpline operators will assess the urgency of symptoms and refer callers to the most appropriate healthcare provider such as the ED or general practitioner. In addition to triage for serious pathology, the helpline service will include ongoing assessment to identify callers who may not be suitable for helpline-based management alone. This may include individuals with multiple psychosocial impairments, poor mental health, progressing symptoms or minimal improvement in patient-reported outcomes. Where appropriate, helpline operators may assist in locating and/or facilitating contact with the local healthcare services to ensure timely and comprehensive management.

A detailed description of the codesign process, components included in the helpline upgrade, and the media campaign will be presented in a separate publication following the development and implementation of the upgraded helpline service.

### Evaluation

The RE-AIM framework^37^ will be used to evaluate the implementation and effectiveness of the upgraded helpline service. The framework includes five dimensions (RE-AIM) used to understand and evaluate the broader impact of the implementation and intervention across individual, setting and system levels (see table 1). PRISM^36^ will be used as an extension of the RE-AIM framework to guide the exploration of the contextual factors that influence the project outcomes. This includes the characteristics and perspectives of recipients (eg, helpline operators, stakeholders and users), the implementation and sustainability infrastructure (eg, helpline operator capacity vs caller demand), and external environment related to long-term sustainability and scalability of the helpline (eg, government funding and policies for primary care). It can also be used to understand and guide necessary adaptations to improve the intervention implementation process.^49^

**Table 1:** Evaluation of implementation and effectiveness using the RE-AIM framework

Evaluation outcome	Study design	Data source	Time points	Outcome measure	Data analysis	Sample size
Reach						
Reach of the media campaign Reach of the helpline	Cross- sectional Interrupted time series	Population panel surveys Monthly helpline call numbers	(1) Pre–post media campaign (2) 18 months pre to 12 months postmedia campaign	Comparison of helpline awareness between two Local Health Districts LBP monthly call rates	χ² test for proportions Segmented regression	700 panel survey participants (post campaign) All LBP phone calls/services received
Effectiveness						
ED visits for LBP (population level) Self-reported healthcare service utilisation (individual level)	Interrupted time series Independent pre–post cohort design	All LBP ED visits recorded in the Local Health District targeted by the media campaign Pre/Post helpline cohort self-reported data.	18 months pre–post upgrade Baseline, 3 months for each cohort	Monthly LBP ED visits Self-reported healthcare service utilisation	Segmented regression Negative binomial regression for count of healthcare service utilisation	All adult ED LBP visits registered with corresponding health district database 295 cohort participants
Adoption						
Characteristics of helpline users (eg, different geographical regions and age groups)	(1) Mixed methods	Monthly helpline call numbers Cohort participant interviews	Continuous 18 months pre–post helpline upgrade Pre–post campaign period	LBP monthly call rates stratified by demographic Qualitative study using PRISM framework	Segmented regression with stratification Mixture of inductive and deductive thematic interview analysis	All LBP phone calls/services received 15–20 cohort participants
Implementation						
Fidelity of the upgrade helpline service to fidelity checklist Participants perceived acceptability of the helpline	(1) Process evaluation using mixed methods	(1) Data audit of provided services (2) Cohort self-reported data	Continuous data collection following the upgrade Pre–post cohort	Fidelity checklist TFAQ acceptability questionnaire	Implementation fidelity Descriptive (visual) analysis	20% random audit of all LBP services provided As per effectiveness outcome
Maintenance						
Sustainability of the upgraded helpline service	(1) Mixed methods	Purposive sample for qualitative study Monthly calls in post campaign period and individual effectiveness at 6 months.	Post campaign period for qualitative data (routine data will be continuous) Post campaign	Qualitative study using PRISM framework Proportion of calls post media campaign completion	Mixture of Inductive and deductive interview analysis Segmented regression and negative binomial regression for count of healthcare service utilisation	15–20 cohort participants, 3–10 participants per stakeholder group All LBP phone calls/services

ED, emergency department; LBP, low back pain; PRISM, Practical Robust Implementation Sustainability Model; RE-AIM, Reach, Effectiveness, Adoption, Implementation and Maintenance; TFAQ, Theoretical Framework of Acceptability Questionnaire.

#### Reach

Reach will be assessed to determine the extent to which the media campaign raises awareness of the helpline and influences service utilisation. This will be evaluated using two approaches:

(1) To evaluate media campaign reach, we will conduct population-based surveys in the ‘intervention’ region (the Mid North Coast Local Health District of NSW) targeted by the campaign, and a ‘control’ region (Southern NSW Local Health District) that will remain unexposed to the media campaign. The control region was chosen for its similarity in population size and distribution to the intervention region, as well as the geographical distance of approximately 450 km and different media markets, which are expected to minimise campaign crossover. Surveys will be administered online via a commercial panel company. The survey will include a single campaign awareness question assessing recognition of the helpline service along with basic aggregated demographic information (eg, age group, sex and postcode) to enable region comparisons. Surveys will be conducted at baseline (precampaign) and postcampaign to measure population awareness of the helpline. Sample size calculations are based on the postcampaign comparison between the two regions (baseline awareness will be summarised descriptively). Based on feasible sample sizes provided by the panel company, a total of 300 respondents in the intervention region and 400 in the control region will be surveyed at both time points. Assuming 0.05% of respondents are aware of the helpline in the control group, this provides 85% power to detect a 3% difference in awareness between regions at a two-sided significance level of 5%.

(2) To evaluate the impact of the campaign on service use, we will analyse the monthly call rates using retrospective data to compare the rates of unscheduled calls with a primary complaint of LBP. This will be analysed 18 months before and 12 months after the campaign begins. An interrupted time series design will be used to determine the effect of the media campaign on call volume.

#### Effectiveness (primary intervention outcome)

The effectiveness of the helpline service will be evaluated at both an individual participant and population (health district) level.

Participant-level effectiveness will be evaluated using a pre–post cohort study. The primary outcome to assess individual effectiveness will be self-reported healthcare use (eg, general practitioner and imaging usage). This will be compared between an independent cohort of participants with a primary complaint of LBP who use the original service, and an independent cohort who use the upgraded service. The primary outcome will be analysed 3 months post service initiation. Cohort participant questionnaires will also capture secondary outcomes including pain intensity,^50^ disability,^51^ quality of life,^52^ the validated single-item physical activity question,^53^ two-item pain self-efficacy,^54^ health confidence score^55^ and acceptability.^41^ Based on historical service volume data from the MHA helpline, we anticipate approximately 50 participants in the preupgrade period and will require a minimum of 208 participants in the postupgrade period to detect an event rate difference of 4 healthcare service utilisations between the preupgrade and postupgrade cohort, with 80% power and 5% type-1 error rate. This is the equivalent of a 40% difference based on an average health service utilisation rate of 10 services per participant over 12 months observed in unpublished work.^56^ Adjusting for a loss to follow-up of 15% in the postupgrade cohort, we will require 245 participants postupgrade to achieve the required 208 completers. Combined with the anticipated preupgrade cohort, the total sample size is 295 participants.

Population effectiveness will be evaluated using aggregated Local Health District data on ED presentations for patients with a complaint of LBP. The analyses will be restricted to the Mid North Coast Local Health District (or relevant hospitals) data corresponding with the location of the media campaign. An interrupted time series design will be used to analyse the monthly change in LBP presentation rates and any associated trend change following the introduction of the media campaign.

#### Adoption

Adoption of the helpline will evaluate the equitable use of the service by examining user representation across different patient demographics. This will be assessed using monthly number of unscheduled callers with a primary complaint of LBP across different demographics (eg, age, sex, rurality—determined using participants’ postcode). An interrupted time series design stratifying by participant demographic will be used to analyse this. Quantitative results will be complemented by one-on-one qualitative interviews with approximately 15–20 participants (pending data saturation) from the pre–post cohort study. Interviews will be conducted online and are estimated to take between 45 and 60 min. Results will be combined to understand the type of patients using the service as well as potential barriers and facilitators to utilisation. Adoption evaluation will inform changes to the intervention, media campaign or infrastructure that may be needed to improve equitable service access.

#### Implementation

To determine if the upgraded helpline is being implemented as intended, a fidelity checklist will be codeveloped with helpline operators and research team ensuring it aligns with the 2022 Australian LBP Clinical Care Standards.^13^ A random audit of 20% of all LBP episodes of care provided following the helpline upgrade will be conducted. Deidentified transcripts of recorded phone calls will be analysed to review the main topics discussed, information and assistance given, and follow-up plans, in accordance with the codeveloped fidelity checks. The proportion of audited calls will be rated as poor, average, good or excellent (based on the codesigned checklist and codefined criteria for each rating). We will also investigate whether there is any association between the fidelity of calls and the time/day the service was delivered or demographics of the callers. In addition, any adaptations to the service or data collection will be recorded and reported. This information will be combined with user-rated acceptability of the helpline, captured as part of the pre–post cohort study questionnaires. A modified version of the TFAQ will evaluate participants’ acceptability of the helpline across several domains including affective attitude, burden, ethicality, intervention coherence, opportunity costs, perceived effectiveness and self-efficacy.^41^

#### Maintenance

At an individual level, maintenance will be evaluated as the impact of the upgraded helpline on self-reported healthcare service use, 6 months following initial contact with the helpline. This will be captured as part of the pre–post cohort study and analysed as per the individual effectiveness outcome. The proportion of callers reporting repeat use (either for the same LBP episode or a new episode) of the helpline service will also be descriptively reported. In addition, cohort participants’ confidence in self-care, measured broadly by the Health Confidence Score,^55^ will be evaluated at 6 months following initial contact. The Health Confidence Score asks participants to rate their knowledge about their health, their ability to self-manage, their ability to navigate the healthcare system and their involvement in decision-making.

At a service level, we will evaluate the sustainability of the upgraded helpline using a mixed-methods approach. This will combine information from qualitative interviews conducted with the cohort participants and two stakeholder focus groups (helpline operators and health service providers), with administrative data on helpline operations (eg, service costs). A minimum of two 1-hour online focus group meetings will be conducted, one for each stakeholder group. Focus groups will explore facilitators and barriers to uptake, effectiveness and sustainability of the helpline.

Cost-effectiveness will be analysed from a health-system perspective utilising the quality-adjusted life-years, based on utility weights from the Assessment of Quality of Life 4D questionnaire^52^ administered at baseline, 3-month and 6-month follow-ups in the preupgrade and postupgrade cohorts. Costs will be identified from self-reported health service use, helpline costs (eg, staff and infrastructure costs) as well as media campaign costs (eg, content development and advertising).

The qualitative insights, stakeholder consultations and cost-effectiveness data will be combined to evaluate the sustainability of the helpline beyond the lifecycle of the research funding. Additionally, the evaluation will establish the feasibility of this model to be scaled up through a broader media campaign (eg, state or national based campaign) and greater integration into Australian primary care.

### Data collection and management

Evaluation of this implementation-effectiveness project will require data collection from two main sources: (1) primary data collected from participants and (2) secondary de-identified data collected from external providers, including MHA’s electronic records, state-based data custodians (eg, NSW Centre for Health Record Linkage) and the panel survey company. Secondary data will be stored and analysed in accordance with the requirements of external data providers.

#### Participant-level data

All participant-reported data collection will be conducted externally to MHA by the research team. Data collected from participants will include baseline demographic data (eg, age, sex, education level) from both the preupgrade and postupgrade cohorts, as well as the individuals participating in qualitative interviews or focus groups. For a full list of participant collected data and timepoints, see online supplemental appendix A.

Cohort questionnaires will be administered at baseline (within 10 days of initial helpline service utilisation), 3 and 6 months postinitial service utilisation. All participant collected data will be automatically assigned a unique study identification code and stored and managed using REDCap electronic data capture tools hosted at the University of Sydney.^35 57^ Qualitative data collected during interviews, including transcripts of interviews, will be stored in a password-protected folder in the highly protected SharePoint folder hosted by the University of Sydney. Only designated research team members will have access to this data.

#### Secondary data

ED population-level data: Deidentified, aggregated data on monthly ED presentations for LBP among patients aged ≥18 years will be obtained from the Mid North Coast Local Health District. Presentations will be identified using the International Classification of Diseases 9th and 10th editions and Systematized Nomenclature of Medicine-Clinical Terminology (SNOMED CT) diagnostic codes adapted from Anderson *et al*^58^ (see online supplemental appendix B). Data will be extracted through the Emergency Department Data Collection via the NSW Centre for Health Record Linkage. Extraction will cover the 18 months before and after the helpline upgrade. Panel data: Deidentified and aggregated population awareness of the helpline will be collected via panel surveys. These will be administered by a panellist provider premedia and postmedia campaign and stored on the University of Sydney’s highly secure SharePoint site. MHA data: Deidentified demographic data routinely collected by MHA, including aggregated information on age, sex, physical activity, pain severity and area of residence, will be compared with demographic data of the cohorts to assess for selection bias.

### Data analysis

Quantitative data will be analysed to evaluate the reach, effectiveness, adoption and implementation dimensions of the RE-AIM framework. Descriptive statistics will summarise demographics and key characteristics of helpline callers, cohort participants, campaign survey respondents and individuals presenting to the Mid North Coast Local Health District ED with LBP. Descriptive statistics will also be used to summarise the fidelity of the delivered helpline service, with the proportion of total calls rated as poor, average, good or high. Participant-reported acceptability will be summarised visually using stacked horizontal bar charts to display the proportion of respondents selecting each response category across the seven acceptability domains.

A χ² test will be used to compare the postcampaign proportion of individuals aware of the helpline between the control region and the intervention region (exposed to the media campaign). Data collected prior to the campaign will be used to provide a descriptive comparison of baseline awareness between the two regions. If notable baseline differences in awareness are identified, a logistic regression will be conducted to estimate the association between the region (intervention or control) and postcampaign awareness, adjusting for baseline awareness as a covariate. An interrupted time series with segmented regression and a generalised Poisson or negative binomial model (dependent on data overdispersion) will be used to evaluate the impact of the media campaign (eg, the interruption point)^59^ on the monthly helpline call rate, the monthly helpline call rate stratified by age, sex and rurality demographics, and the monthly rate of ED presentations for LBP within the campaign exposed Local Health District. Exploratory analyses will be conducted to inspect visual trends, with adjustments for autocorrelation and seasonality conducted prior to final analysis. The estimated impact of the media campaign on each outcome, for example, an immediate (stepped change) or trend change (slope change) will be prespecified in the statistical analysis plan once the final media strategy has been determined.

A comparison of primary participant-reported outcome (self-reported healthcare service utilisation) and secondary outcomes, between the preupgraded and postupgraded helpline cohorts will be conducted. The primary outcome will be analysed at 3 months (as part of the effectiveness dimension) and 6 months (as part of the maintenance dimension). Healthcare service use will be collected as count data and analysed using negative binomial regression model. The rate ratio will be estimated comparing the postupgrade cohort to preupgrade cohort. Negative binomial regression will account for individual follow-up time and potential overdispersion due to non-independence of repeated measures. Matching with replacement will be used to address potential imbalances between cohorts in baseline demographics and to account for the anticipated smaller ‘unexposed’ preupgrade cohort compared with the larger ‘exposed’ postupgrade cohort. Sensitivity analyses will be conducted to assess the robustness of the findings.

Individual qualitative interviews will be conducted with purposively selected cohort participants to identify factors and processes that contributed to the helpline uptake, implementation and future scalability. These include acceptability and appropriateness (the extent to which the helpline is perceived as agreeable and relevant); advertising campaign effectiveness; individual factors (eg, participants’ socioeconomic status); and service outcomes (eg, the extent to which the helpline is perceived to be effective, patient centred, timely). Interviews will be semistructured, using a mixture of inductive and deductive thematic analysis drawing on the PRISM framework.^36^ An interview guide will be used to ensure the same topics are explored across individual interviews.

Focus groups with key stakeholder groups will be conducted online to identify factors and processes that contributed to helpline uptake, implementation and future scalability. The interview guide will be informed by findings from the implementation phase to align with observed barriers and enablers. Focus group data will be analysed using a combination of inductive and deductive approaches, drawing on the PRISM framework to guide interpretation.

## Ethics and dissemination

This project will be completed in accordance with the guidelines set out in the Australian National Statement on Ethical Conduct in Human Research.^60^ Ethics approval for the helpline project has been provided by the University of Sydney’s Human Research Ethics Committee (Ethics Approval HE001081). This study does not assign participants to an intervention or treatment but collects data on outcomes following the use of a freely available, and nationwide helpline service. Nonetheless, this study has been designed to minimise and prevent any harm or risks to potential study participants. Adverse events will be monitored throughout the study. Cohort participants will be prompted fortnightly (via text or email according to preferences) to report any adverse events in the preceding 2 weeks via a REDCap survey. Study findings will be disseminated through peer-reviewed research publications and conference presentations, as well as via our partner organisations and involved consumer-advocacy groups. A deidentified, curated data set (excluding data collected from external providers) may be placed in a secure open access repository in accordance with National Health and Medical Research Council (NHMRC) best practice.^61^ In the case of repository data, the chief investigator will ensure the shared data meet’s both the participants’ consent agreement and Multi-Institutional Agreement requirements.

## Discussion

This protocol describes the implementation and evaluation plan for the helpline project, which may inform future management of LBP in Australia and contribute evidence to guide national implementation. Using an iterative co-design approach, this project aims to develop and evaluate an upgraded helpline that delivers LBP care aligned with stakeholder objectives, while meeting consumer needs. The RE-AIM framework with PRISM extension will enable evaluation of the effectiveness of the intervention and implementation process alongside the contextual factors that influence outcomes. Guided by these frameworks, the research findings are expected to inform the infrastructure required to sustain the helpline service, and the feasibility of the model to support greater call numbers through a broader media campaign.

Anticipated outcomes include improved awareness and uptake of the helpline service, and delivery of evidence-based, patient-centred care that may reduce unnecessary healthcare utilisation. The success of the service is dependent on several factors including the reach of the media campaign to promote awareness, operator fidelity to the codesigned protocol and the ability of the helpline to meet diverse patient needs.

This study also has potential limitations. Promotion and uptake of the service observed across one Local Health District may not be generalisable to other regions of Australia, and increased uptake of the helpline service following the media campaign may not translate to sustained service use once the campaign concludes. To preserve MHA’s aims of providing a freely available nationwide helpline service, the type of design that could be employed to test effectiveness was limited. While a pre–post cohort design allows comparison of service effectiveness before and after the upgrade, the absence of a randomised control group restricts the ability to draw definitive causal inferences.

Finally, individual variability needs and treatment responses among people with LBP mean it is unlikely that the helpline will be equally effective and acceptable for all users. However, implementation evaluation will help identify factors influencing uptake and inform future adaptations to improve reach and acceptability, and effectiveness of the LBP helpline service.

## Supplementary material

10.1136/bmjopen-2025-106605 online supplemental file 1

10.1136/bmjopen-2025-106605 online supplemental file 2
