# Supplementary material for: Implementation of an Australian helpline for low back pain: protocol of a type 2 hybrid effectiveness-implementation trial
Source: BMJ Open. 2025 Dec 2;15(12):e106605. doi: 10.1136/bmjopen-2025-106605 (PMC12673535; doi:10.1136/bmjopen-2025-106605)
Supplement: online supplemental file 1 [file bmjopen-15-12-s001.docx]

***Outcome Measures***

*Table 2. Outcome Measures collected*

| **Measure** | **Method** | **Description** | **Scale** | **Timepoint** |
| --- | --- | --- | --- | --- |
| **Data collected directly from participants** | | | | |
| **Demographics** | | | | |
| **Date of birth** | Self-reported | Please enter your date of birth | DD/MM/YYYY | Baseline |
| **Sex** | Self-reported | What is your sex? | 1= Male  2= Female  3= Other  4= Prefer not to say | Baseline |
| **Location** | Self-reported | What is your postcode? | 4 digit number | Baseline |
| **Language used at home** | Self-reported | What language do you use at home? | 1= English  2=Other; please specify | Baseline |
| **Education level** | Self-reported | What is the highest level of schooling you have completed? | 0 =None  1=Primary  3= Secondary  4=Tertiary (TAFE/Apprentice)  5= Tertiary (e.g., University Bachelor) | Baseline |
| **Current Employment Status** | Self-reported | What is your current employment status? | 1 = Working full-time  2 = Working part-time  3 = Unable to work at the moment  4 = Retired  5 = Unemployed  6 = Homemaker  7 = Student | Baseline |
| **Co-morbid conditions** | Self-reported | Do you have a current health condition or disease, as diagnosed by a medical doctor? | 1 = No  2 = Yes  If yes, please, specify: | Baseline |
| **Duration of low back pain** | Self-reported | How long have you experienced low back pain? | 1 = <4 weeks.  2 = Between 4-12 weeks  3 = Between 12 weeks and 1 year.  4= More than 1 year, please specify how many years: | Baseline |
| **Characteristics of low back pain symptoms** | Self-reported | Regarding your low back pain, which best describes your symptoms? | 1 = Back pain only.  2 = Back pain with leg pain.  3 = Leg pain only | Baseline |
| **Patterns of low back pain** | Self-reported | Regarding your low back pain, which best describes your symptoms? | 1 = Constant back pain (always present and never fully recovers).  2 = Recurrent back pain (intermittent episodes of back pain).  3 = Other, please specify? | Baseline |
| **Surgery for back pain** | Self-reported | Have you ever had surgery for your low back pain | 1= No  2= Yes  If Yes, what type of surgery/surgeries have you had? | Baseline |
| **Primary Outcome** | | | | |
| **Healthcare Use** | Self-reported Health Service utilisation survey. | Bespoke designed survey to provide self-reported use of any hospital services (e.g., ED), outpatient services, community-based health practitioners, diagnostic tests (e.g., imaging), and medications for LBP. Questions refer to the past month of use. | Self-reported healthcare service utilisation will be defined as the number of healthcare provider visits and diagnostics tests or procedures used. Type, frequency and dosage of medications used, will also be captured through self-report data. See Help Line Project Study Tools-baseline questionnaire. | Baseline (following use of the Help Line service), 3- and 6-months post baseline completion. |
| **Secondary Outcomes** | | | | |
| **Physical Activity Levels** | Single-Item physical activity measure | ﻿Single item questionnaire asking; “In the past week, on how many days have you done a total of 30 minutes or more of physical activity, which was enough to raise your breathing rate. ﻿This may include sport, exercise, and brisk walking or cycling for recreation or ﻿to get to and from places but should not include housework or physical activity that may be part of your job”. Participants will select the response that best matches their activity level. | The response scale will be from 0 to 7 days. | Baseline (following use of the Help Line service), 3- and 6-months post baseline completion. |
| **Pain Intensity** | Numeric Rating Scale | The NRS is a single-question, unidimensional self-reported instrument designed to assess pain intensity in adults, including those with low back pain.  Participants rate their average pain intensity in the last 24 hours and in the preceding week. | Pain intensity is rated on an 11-point numerical rating scale ranging from 0 = “no pain” to 10 = “worst pain imaginable”. | Baseline (following use of the Help Line service), 3- and 6-months post baseline completion. |
| **Disability** | Roland-Morris Disability Questionnaire (RMDQ) | The RMDQ, originally proposed by Roland and Morris, is a 24-item, valid, reliable, and responsive self-reported instrument designed to assess disability associated with activities of daily living in people with low back pain.  Participants are asked to check mark the statements that apply to them that day. | Each statement with a check mark is worth 1 point.  The total score, calculated by summing the scores for each statement, ranges from 0 to 24, where “0” indicates “no disability” and “24” indicates “extremely severe disability”. | Baseline (following use of the Help Line service), 3- and 6-months post baseline completion. |
| **Quality of Life** | Assessment of Quality of Life questionnaire (AQoL-4D) | The AQoL instrument is a multi-attribute quality utility, health related quality of life instrument. It is used to measure quality of life and is suitable for use in economic evaluations. Participants are asked to mark the statements that best described their situation over the past week. | The AQoL-4D has 12 questions which can be weighted to inform economic evaluations | Baseline (following use of the Help Line service), 3- and 6-months post baseline completion. |
| **Acceptability** | Generic Theoretical Framework of Acceptability (TFA) Questionnaire | The TFA acceptability questionnaire consists of 9 items: one item reflecting each of the TFA constructs. It assesses the acceptability of healthcare interventions. Participants are asked to mark the statements that best reflect their opinion of the Help Line intervention. | 8 items scored on a scale of 1 to 5, individualised for each construct of acceptability. | Baseline (following use of the Help Line service), 3- and 6-months post baseline completion. |
| **Pain Self-Efficacy** | Pain Self-Efficacy Questionnaire-Two-Item Short Form (PSEQ-2) | The PSEQ-2 is the short form questionnaire of the widely used 10-item Pain Self-Efficacy Questionnaire, used to measure self-efficacy in people with chronic pain. | 2 items rated on a scale of 0 to 6. | Baseline (following use of the Help Line service), 3- and 6-months post baseline completion. |
| **Health Confidence** | Health Confidence Score questionnaire. | The HCS is a four-item questionnaire designed to assess an individual’s health management/self-care confidence. | There are four items that are scored using the following scale; strongly agree, agree, neutral, disagree. | Baseline (following use of the Help Line service), 3- and 6-months post baseline completion. |
| **Qualitative Data**  **including demographics:**  Age  Sex  Education  Occupation  Postcode  Role in Help Line (i.e., user, operator, referrer, partner provider) | Mixture of one-on-one interviews (for users of the Help Line), and small groups for the Co-design Consumer and stakeholders focus groups. | 1) Nominal Group Technique, 2) one-on-one and focus groups using inductive and deductive thematic analysis drawing on the PRISM framework. | N/A | Data collected in pre- and post-campaign period. |
| **Data collected from MHA health records (deidentified)** | | | | |
| **Demographics** | | | | |
| **Age** | Self-report | Please enter your age | Years | Routine questions administered at the beginning of the Help Line call |
| **Sex** | Self-report | What is your sex? | 1= Male  2= Female  3= Other  4 = Prefer not to say | Routine questions administered at the beginning of the Help Line call |
| **Physical Activity** | Self-report- Single-Item physical activity measure | ﻿ Single item questionnaire asking; “In the past week, on how many days have you done a total of 30 minutes or more of physical activity, which was enough to raise your breathing rate. ﻿This may include sport, exercise, and brisk walking or cycling for recreation or ﻿to get to and from places, but should not include housework or physical activity that may be part of your job”. Participants will select the response that best matches their activity level. | The response scale will be from 0 to 7 days. | Routine questions administered at the beginning of the Help Line call |
| **Pain** | Self-report Numeric Rating Scale | The NRS is a single-question, unidimensional self-reported instrument designed to assess pain intensity in adults, including those with low back pain.  Participants rate their average pain intensity in the last 24 hours and in the preceding week | Pain intensity is rated on an 11-point numerical rating scale ranging from 0 = “no pain” to 10 = “worst pain imaginable”. | Routine questions administered at the beginning of the Help Line call |
| **Postcode** | Self-reported | What is your postcode? | 4-digit number | Routine questions administered at the beginning of the Help Line call |
| **Data collected from external sources: State based data custodians (e.g., NSW-CHeReL) and survey administrators** | | | | |
| **Emergency Department presentation for low back pain** | ED data will be extracted using state-based data-custodians. ICD and SNOMED diagnostic codes will be used to identify back pain presentations. | Monthly counts of ED presentations for low back pain will be extracted. | Count data. | Monthly count data will be extracted 18 months prior to the upgraded Help Line, and 18 months post the upgraded Help Line. |
| **Awareness of the MHA’s national Help Line**  Mean/median age of respondents, proportion of males vs female | Panel surveys will be utilised to conduct population-based surveys to determine the awareness of the Help Line in two Local Health Districts | An additional question will be added to the regularly conducted survey asking Q1“Are you aware of Musculoskeletal Health Australia’s B.A.M Helpline?” | Q1-Survey respondents will mark either Yes or No. | Surveys will be conducted prior to the campaign and in the post campaign period. |
