# Supplementary material for: Implementation of an Australian helpline for low back pain: protocol of a type 2 hybrid effectiveness-implementation trial
Source: BMJ Open. 2025 Dec 2;15(12):e106605. doi: 10.1136/bmjopen-2025-106605 (PMC12673535; doi:10.1136/bmjopen-2025-106605)
Supplement: online supplemental file 2 [file bmjopen-15-12-s002.docx]

Table 3. Emergency Department Low Back Pain diagnostic code set for data extraction

| **Variable** | **Codes** |
| --- | --- |
| Clinical code set | Adapted from Anderson et al 2022:  **International Classification of Diseases (ICD), Ninth revision**, Clinical Modification (721.3 (Lumbosacral spondylosis without myelopathy), 722.1 (Displacement of lumbar intervertebral disc without myelopathy), 722.32 (Schmorl’s nodes, lumbar region), 722.52 (Degeneration of lumbar or lumbosacral intervertebral disc), 722.93 (Other and unspecified disc disorder, lumbar region), 724.02 (Spinal stenosis, lumbar region, without neurogenic claudication), 724.03 (Spinal stenosis, lumbar region, with neurogenic claudication), 724.2 (Lumbago), 724.3 (Sciatica), 724.5 (Backache, unspecified), 724.6 (Disorders of sacrum), 738.5 (Other acquired deformity of back or spine), 739.3 (Nonallopathic lesions, lumbar region), 739.4 (Nonallopathic lesions, sacral region), 846 (Sprain of lumbosacral (joint) (ligament)), 846.1 (Sprain of sacroiliac ligament), **ICD Tenth revision**, Australian Modification (M48.4 (fatigue fracture of vertebra); M48.5 (collapsed vertebra, not elsewhere classified), M48.0 (spinal stenosis), M51.1-9 (lumbar and other intervertebral disc disorders with radiculopathy), M51.2 (lumbago due to displacement of intervertebral disc), M53.2 (spinal instabilities), M54.3-5 (sciatica), M54.4 (lumbago with sciatica), M54.5 (low back pain). and **SNOMED CT** (161894002 (Complaining of low back pain (finding)), 202794004 (Lumbago with sciatica (finding)), 247368002 (Posterior compartment low back pain (finding)), 267067009 (Lumbar ache – renal (finding)), 267982002 (Pain in lumbar spine (finding)), 275316003 (On examination – lumbar pain on palpation (finding)), 278860009 (Chronic low back pain (finding)), 278862001 (Acute low back pain (finding)), 279039007 (Low back pain (finding)), 279040009 (Mechanical low back pain (finding)), 279041008 (Lumbar trigger point syndrome (finding)), 279042001 (Lumbar segmental dysfunction (finding)), 279063004 (Lumbar facet joint pain (finding)), 298674008 (Lumbar spine – painful on movement (finding)),  300957005 Postural low back pain (finding), 301408007 (Tenderness of left lumbar (finding)), 301407002 (Tenderness of right lumbar (finding)), 298674008 (Lumbar spine painful on movement (finding)), 161891005 (Backache (finding)). |
