# Supplementary material for: Implementation of an Australian helpline for low back pain: protocol of a type 2 hybrid effectiveness-implementation trial
Source: BMJ Open. 2025 Dec 2;15(12):e106605. doi: 10.1136/bmjopen-2025-106605 (PMC12673535; doi:10.1136/bmjopen-2025-106605)
Supplement: Supplementary file 3 [file bmjopen-2025-106605.reviewer_comments.pdf]

PEER REVIEW HISTORY

BMJ Open publishes all reviews undertaken for accepted manuscripts. Reviewers are asked to complete a checklist review form and are provided with free text boxes to elaborate on their assessment. These free text comments are reproduced below.

ARTICLE DETAILS

Title (Provisional)

Implementation of an Australian helpline for low back pain: Protocol of a Type 2 Hybrid Effectiveness-Implementation trial

Authors

Zouch, James; Roberts, Katharine; Bauman, Adrian; Jentz, Helen; Ho, Emma K; Hodges, Paul; Maher, Chris; Baysari, Melissa T; Thompson, Julia; Calder, Rosemary; Luscombe, Georgina; Ceprnja, Dragana; Maka, Katherine; Tian, Ye; Chen, Yanyu; Chen, Michelle; Mork, Paul Jarle; Li, Qiang; Wise, Sarah; Gilbert, Melissa; Hall, Michelle; Ferreira, Manuela L; Ferreira, Paulo

---

VERSION 1 - REVIEW

**Reviewer**	**1**
**Name**	**Pires, Diogo**
**Affiliation**	**Instituto Politécnico de Setúbal**
**Date**	**03-Oct-2025**
**COI**	**None**

I appreciated the opportunity to review your manuscript, which has the potential to represent a significant innovation in global healthcare for people with low back pain. I believe the project is methodologically demanding and that there are substantial challenges in the implementation process. However, I have restricted my comments to aspects that may further improve the manuscript, acknowledging that there is little opportunity to make methodological changes to the project at this stage. Well done and good luck!

Introduction

Part of the rationale underpinning the objectives and relevance of the study is based on the fact that healthcare professionals often do not apply the recommended interventions, and that timely access to healthcare remains problematic (paragraphs 2, 3, and 4 of the introduction). However, it was not clear to me how the helpline to be developed could contribute to addressing these issues. As the authors themselves note, the helpline may be particularly useful in providing education and counselling, preventing unnecessary healthcare seeking, and assisting patients in navigating the healthcare system. Therefore, I

would suggest that the rationale of the study be more explicitly centred on the problems that may be addressed through this new helpline (the intervention under investigation), and on the potential underlying mechanisms.

Methods

To be honest, I found it somewhat difficult to follow the description of the project's phases and associated tasks. I think that a timeline/chronogram could be helpful, but reconsidering the structure of the headings and subheadings should also be taken into account. For example, "design" could be separated into "exploration", "design and development", and "testing and refinement", while "implementation" could stand on its own (page 7 and 8). The same applies to the subheading patient and public partnership statement, which may not fit well under the heading "Evaluation".

On page 9 (lines 17–20), it is stated that the service will include a triage component to redirect participants to healthcare in the case of suspected serious pathology. I think the relationship between the helpline and healthcare services (both in situations of serious pathology and in other circumstances where healthcare seeking is desirable) should be described in greater detail. On the same page, I believe the authors intended to refer to Table 1, rather than Table 2, which I was unable to locate in the appendix. I understand that the manuscript is already extensive, but I felt that a brief description of the data analysis was missing. At the very least, reference should be made to what is outlined in Table 1 regarding data analysis, although I find this summary to be overly concise.

VERSION 1 - AUTHOR RESPONSE

Review 1 comments:

1. Introduction

Part of the rationale underpinning the objectives and relevance of the study is based on the fact that healthcare professionals often do not apply the recommended interventions, and that timely access to healthcare remains problematic (paragraphs 2, 3, and 4 of the introduction). However, it was not clear to me how the helpline to be developed could contribute to addressing these issues. As the authors themselves note, the helpline may be particularly useful in providing education and counselling, preventing unnecessary healthcare seeking, and assisting patients in navigating the healthcare system. Therefore, I would suggest that the rationale of the study be more explicitly centred on the problems that may be addressed through this new helpline (the intervention under investigation), and on the potential underlying mechanisms.

Authors response: We agree that the rationale should clearly articulate how the proposed helpline addresses the identified gaps in care and access. The introduction has been revised,

by removing unnecessary information, and focusing on the connection of the helpline's potential functions—accessible education and advice, timely care, and navigation support—to the observed barriers of limited access to timely evidence-based care, lack of public funding for allied health management, and public uncertainty about care pathways. We believe these revisions clarify how the helpline model directly targets the structural factors contributing to suboptimal LBP management.

"…Guideline management recommendations for LBP have remained relatively consistent for 20 years.12 They advocate for a non-pharmacological approach that includes advice and education, self-care, and physical activity. Imaging is recommended only for suspected serious pathology and medications should be used judiciously.13 However, evidence on contemporary LBP management practices indicates a gap between guideline recommended care13 and the care that patients receive.14–16 For example, in primary (first-contact) care settings approximately 25% of patients with LBP are referred for imaging,16 more than one-third are prescribed at least one opioid medication within a year of diagnosis,17 and only half of Australian patients surveyed recalled receiving education and advice.18 In emergency departments (EDs), nearly 70% of patients presenting with LBP receive an opioid medication and almost one-quarter are imaged.19 This discrepancy may reflect inequities in access to high-value care and resources, and inadequate support to self-care.

While considerable research has been directed toward improving clinician adherence to LBP guidelines,20 addressing structural barriers within the Australian healthcare system may be essential to bridging the gap between guideline recommended LBP care, and the care that patients receive. Key barriers include inadequate access to timely and appropriate care,21 limited public funding to support allied health management,22 and affordability of 'out-of-pocket' costs and private health insurance.23 Exacerbating these structural barriers are widespread public misconceptions about the causes of LBP and appropriate management choices. Combined with patients' lack of awareness about the roles and scope of practice of different healthcare providers, this often results in unsupported self-care, uncertainty about where to seek appropriate care for management of their LBP,21 and unnecessary health service utilisation.

The evidence highlights an urgent need to explore alternative strategies to improve LBP care and reduce the widespread impact of LBP on individuals and the healthcare system.2 Through reformed healthcare pathways and funding models, these strategies should focus on improving access to evidenced based information and care, supporting individuals to remain active at work and in daily life, and reducing unnecessary health service utilisation. One potential solution is the use of helplines – telephone-based services that typically provide free access to health information and advice, with the aim of reducing face-to-face

care.24 They overcome traditional barriers to accessing care and provide a platform to disseminate evidence-based educational resources at scale and with little geographical limitation"

Review 1 comments:

2. Methods

To be honest, I found it somewhat difficult to follow the description of the project's phases and associated tasks. I think that a timeline/chronogram could be helpful, but reconsidering the structure of the headings and subheadings should also be taken into account. For example, "design" could be separated into "exploration", "design and development", and "testing and refinement", while "implementation" could stand on its own (page 7 and 8). The same applies to the subheading patient and public partnership statement, which may not fit well under the heading "Evaluation".

On page 9 (lines 17–20), it is stated that the service will include a triage component to redirect participants to healthcare in the case of suspected serious pathology. I think the relationship between the helpline and healthcare services (both in situations of serious pathology and in other circumstances where healthcare seeking is desirable) should be described in greater detail. On the same page, I believe the authors intended to refer to Table 1, rather than Table 2, which I was unable to locate in the appendix. I understand that the manuscript is already extensive, but I felt that a brief description of the data analysis was missing. At the very least, reference should be made to what is outlined in Table 1 regarding data analysis, although I find this summary to be overly concise.

Authors response: We thank the reviewer for this comprehensive and constructive feedback. In response, we have made several revisions to improve clarity and structure:

• A Gantt chart (Figure 1) has been added to illustrate the study timeline and clarify the sequence of project phases.

• The Methods section has been substantially restructured to follow a more traditional format, comprising: Study design; Gantt chart (Figure 1); Setting and stakeholders; Patient and public involvement; Participant recruitment and consent; Design; Implementation; Evaluation; Data collection and management; Data analysis; Ethics and dissemination; and Discussion.

• The Design phase has been expanded and separated into three distinct subsections—Exploration, Design and development, and Testing and refinement—as suggested. The Implementation section now stands alone.

• The Patient and public involvement section has been relocated to improve alignment with recommended reporting standards.

• We have added further detail describing the anticipated relationship (this is part of the co-development process) between the helpline and existing healthcare services, including processes for triaging or redirecting callers when serious pathology is suspected or when additional healthcare input is appropriate.

• A new Data analysis section has been added to outline the analytic approaches for both quantitative and qualitative data, supplementing the summary provided in Table 1.

• In addition, new appendix tables now detail all data sources, collection methods, and time points, along with the low back pain diagnostic codes extracted as part of the secondary data analyses.

We believe these revisions collectively improve the transparency, structure, and readability of the protocol.

VERSION 2 - REVIEW

**Reviewer**	**1**
**Name**	**Pires, Diogo**
**Affiliation**	**Instituto Politécnico de Setúbal**
**Date**	**06-Nov-2025**
**COI**	

I appreciate the authors' effort to address my questions and suggestions and to improve the manuscript accordingly. I believe my concerns have been adequately addressed. Best of luck with this ambitious project.
